# We Must Consider a Growing Opioid Epidemic in Older People

**DOI:** 10.1177/21514593221083823

**Published:** 2022-03-09

**Authors:** Murray A J Hudson, Louis J Koizia, Michael B Fertleman

**Affiliations:** Cutrale Perioperative and Ageing Group, Department of Bioengineering, 4615 Imperial College London, White City Campus, Sir Michael Uren Hub, 86 Wood Lane, London W12 0BZ, UK

**Keywords:** older people, geriatric, opioids, opioid epidemic

Dear Sir,

We are enthused to read Davies et al (2022)^1^ recent series suggesting that transdermal buprenorphine is safe and effective in the management of pain following a neck of femur fragility fracture. Their paper brings to light an area of broader research which demands attention; the *safe* use of opioids in older people. Whilst adequate pain relief is critical for the older person, it is our growing fear that there is a general sense amongst geriatricians that we are immune to the iatrogenic opioid epidemic. It is recognised that the number of opioid prescriptions in older patients has increased dramatically, and strong opioid prescribing is increasing at the fastest rate in older people.^2^ The international opioid crisis is well discussed and documented, but almost exclusively in younger adults. A systematic review noted a dearth of high-quality research on prescription drug misuse in older adults,^3^ and Mikelyte et al (2020)^4^ highlights the gap in understanding the scope of opioid prescribing in older people. Opioids contribute to older people experiencing falls, memory problems and incontinence. Opioids are the fifth most likely drug class to cause preventable hospital admissions,^5^ and the adverse effects occur more frequently in the older population.^6^ Alongside these concerns, work in the community found that of older people on low-dose opioid analgesics for a year, 40% met ICD–10 criteria (World Health Organization 1992) for dependence syndrome.^7^ A recent study found 10% of in-patients over 65 were opioid dependent.^8^

Strides have been made in the understanding and more careful use of benzodiazepines in older people. Whilst the opioid epidemic appears to garner an even greater public awareness, as geriatricians, we must act now to stem its potentially huge impact on that older population. Davies et al (2022) have considered strong opioid analgesia use in hospital, and despite their practice of buprenorphine patch removal on discharge (or at 2 weeks), they have not included information on analgesia requirements following discharge from hospital when the patch is removed. On a local level, we recognised the increasingly large number of older patients being prescribed opioids during their admission. We found that this continued to discharge and at 120 days post discharge. As a result of our findings, we have targeted our efforts on avoiding unnecessary and/or combined prescriptions of stronger opioids. We instigated senior stewardship over prescribing, reviewing analgesics daily and aimed to avoid use of opioids at all on transfer from hospital to intermediate or community care. Whilst we welcome Davies et al (2022), it remains our responsibility as geriatric clinicians to do much more to evaluate and ‘control’ the appropriate and safe use of strong opioids in older people. We must support national bodies and agencies to stem the opioid epidemic that is now impacting this older population.
